# Dark Matter Transcripts: Sound and Fury, Signifying Nothing?

**DOI:** 10.1371/journal.pbio.1000370

**Published:** 2010-05-18

**Authors:** Richard Robinson

**Affiliations:** Freelance Science Writer, Sherborn, Massachusetts, United States of America

**Figure pbio-1000370-g001:**
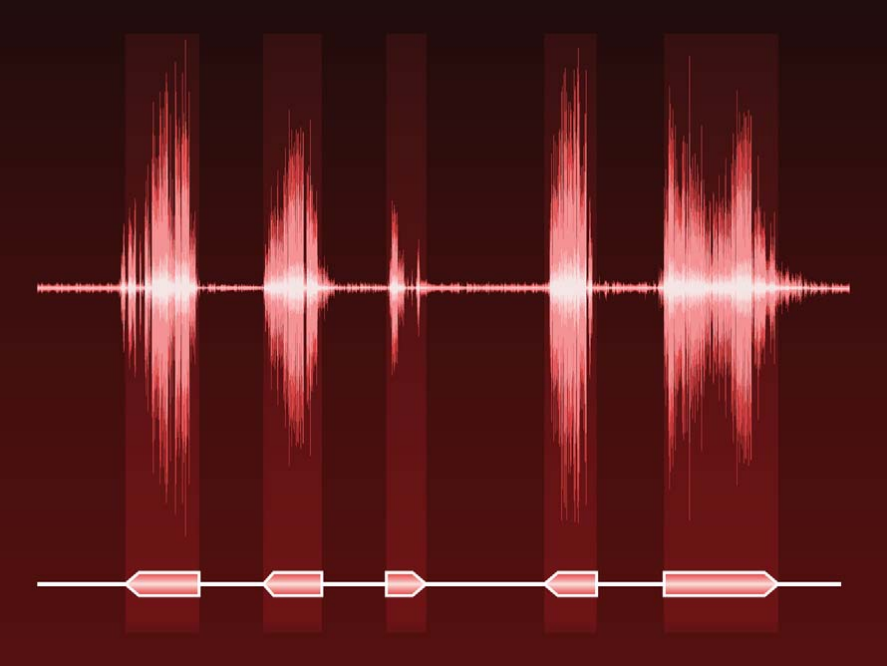
Transcript profiles around genes are rendered as a sound wave.

[Fig pbio-1000370-g001]It seems, in retrospect, that understanding the protein-coding portion of the genome was the easy part; it's the other 98% that's the real challenge. Once derided as mere “junk DNA”—the useless relics of ancient mistakes—the non-coding regions recently earned a great deal more respect, stemming from a series of reports that these regions were hotbeds of transcription. The abundance of RNA signals from this mysterious genomic “dark matter” appeared to indicate that the genome was up to a whole lot more than simply churning out proteins from well-described genes.

But a new study published in this issue of *PLoS Biology* by Harm van Bakel, Timothy Hughes, and colleagues shows that most dark matter transcripts are likely to be by-products of transcription of known genes and that many of the rest of them are likely not messages of great import, but simple background noise.

The earlier reports detected dark matter transcripts using “tiling arrays,” DNA microarrays embedded with probe sequences drawn from regularly spaced regions across the genome. These reports indicated that a quarter or more of all transcripts created by the nucleus arose from DNA completely outside the boundaries of known genes. The nature and function of these transcripts was unclear, but while some likely arose from spillover transcription of known genes, some from novel genes, and some from erroneous activity of RNA polymerase, it seemed logical, given their apparent numbers, that many others were RNAs with new and unknown function. But data from arrays are prone to false positives—since a probe may bind to a less-than-perfect match when no perfect match is available—leading skeptics to wonder whether many of the signals arising from dark matter are really there at all.

More recently, it has become practical to sample the sequence of large numbers of RNA transcripts, a technique unavailable even a few years ago when dark matter transcripts were first discovered. So, the authors began their study by comparing results in both mouse and human tissues from tiling arrays to those from exhaustive RNA sequencing. They showed convincingly that the sequencing data identified transcription from many fewer non-gene regions, suggesting that much of the tiling data arose from false-positive noise, rather than actual unique RNA sequence signal.

So, if exhaustive sequencing is the right tool for exploring the dark matter of the genome, what does it reveal? First, transcripts from dark matter make up only 12% of all polyadenylated transcripts; the rest arise from exons of well-known genes. In fact, after excluding introns and a couple of other well-described categories of transcripts, only about 2% of all transcripts are left unexplained. Many of these turn out to be fragments transcribed from the tail end of genes, possibly arising as a result of alternative termination sites or when RNA polymerase fails to disengage after transcribing the gene proper. Three quarters of the unexplained RNA sequences arose from within 10 kb of either side of known genes, although these regions make up less than 20% of the intergenic regions. Most of the rest of the unexplained sequences resembled a random distribution in both location and copy number. Similar conclusions were reached by analyzing non-polyadenylated transcripts.

There were nonetheless some intriguing real signals from the vast regions outside of known genes. About 1% of all RNA transcripts, amounting to several thousand distinct sequences, occurred in high enough copy number and, in many cases, in regions conserved between mammals to suggest they were products of active transcription. These predominantly arose in open chromatin—DNA that is unpacked and accessible to RNA polymerase. But, whether the chromatin was open in order to transcribe these sequences, or whether they were transcribed because the chromatin is open for other reasons, is unknown. Neither is there any known function for the transcripts, leaving open the possibility that these, too, are a byproduct of other, more directed RNA polymerase activities.

The emerging picture of RNA polymerase is of an inherently imprecise, not to say promiscuous, copyist, one whose output includes some mistakes along with lots of valuable product. In this view, most dark matter transcripts are not signals emerging from a hidden universe within the genome, but instead simply the noise emitted by a busy machine.

**van Bakel H, Nislow C, Blencowe BJ, Hughes TR (2010) Most “Dark Matter” Transcripts Are Associated With Known Genes. doi:10.1371/journal.pbio.1000371**

